# The human cost of Type-2 diabetes mellitus: Uncovering the hidden burden on quality of life

**DOI:** 10.12669/pjms.41.3.10841

**Published:** 2025-03

**Authors:** Shireen Javed, Mahaneem Mohamed, Benash Altaf, Wan Syaheedah Wan Ghazali

**Affiliations:** 1 Shireen Javed, (MBBS, MPhil, PhD Scholar) Associate Professor, Department of Physiology Aziz Fatimah Medical and Dental College, Faisalabad, Pakistan; 2 Mahaneem Mohamed, (MD, PhD) Associate Professor, Department of Physiology, School of Medical Sciences, Universiti Sains Malaysia, Kelantan, Malaysia; 3 Benash Altaf, (MBBS, MPhil, PhD Scholar), Associate Professor, Department of Physiology Aziz Fatimah Medical and Dental College, Faisalabad, Pakistan; 4 Wan Syaheedah Wan Ghazali, (MD, PhD), Medical Lecturer, Department of Physiology, School of Medical Sciences, Universiti Sains Malaysia, Kelantan, Malaysia

**Keywords:** Form-36 questionnaire, Mental component summary, Physical component summary, Quality of life, Short Type 2 diabetes mellitus

## Abstract

**Objectives:**

To evaluate the impact of Type 2 diabetes mellitus (T2DM) on quality of life (QOL) and compare it with healthy subjects.

**Method:**

This case-control study was conducted at Aziz Fatimah Hospital and Aziz Fatimah Medical and Dental College, Faisalabad, Pakistan, from May 2023 to January 2024. A total of 170 subjects were included in the study through purposive sampling technique. Fasting and random blood glucose levels were measured using an enzymatic colorimetric test. HbA1c was determined with the Bioherms A1C EZ 2.0 Glyco-hemoglobin Test Kit. QOL was assessed using the Short Form-36 version 1 questionnaire. Statistical analysis was performed using SPSS version 26, with p ≤ 0.05 considered significant.

**Results:**

The study included 86 T2DM patients and 84 controls, with a mean age of 49.23 ± 12.59 years. Among the participants, 44.7% were male and 55.3% were female. T2DM patients scored lower in physical functioning, limitations due to physical health, bodily pain and vitality subscale compared to controls (p = 0.003, 0.050, 0.030, 0.000 respectively). Both groups had physical component summary and mental component summary (MCS) scores below 50, indicating impaired QOL. MCS scores were significantly lower in T2DM patients than in controls (p = 0.002). Females scored lower in vitality (p = 0.0005) and bodily pain (p = 0.031). T2DM patients with less than five years of duration had lower SF-36 scores than those with longer duration.

**Conclusion:**

T2DM impairs quality of life, especially in females and those with less than five years of disease duration, who face a greater negative impact.

## INTRODUCTION

Diabetes mellitus (DM) is a global health crisis with major impact on the quality of life (QOL) affecting social, physical, and mental well-being.^1^,^2^ According to the World Health Organization (WHO), Type-2 diabetes mellitus (T2DM) accounts for 90% of all diabetic cases and is one of the leading causes of mortality worldwide.^3^ It has been predicted that global prevalence of diabetes will increase by 370 million in 2030 from 170 million patients in 2000.^3^ Type 1 diabetes is on the rise mostly in high socio-demographic index (SDI) nations, such as Europe and the United States while the incidence and prevalence of T2DM is continuously increasing at alarming rate in middle, and high-middle SDI countries.^4^

Pakistan is also facing rapid increase in T2DM with the prevalence of 26.3% as reported by second National Diabetes Survey of Pakistan (NDSP), 2016-2017.^5^ People with diabetes are often seemed to be associated with impaired QOL due to associated comorbidities and demanding costly management.^3^,^6^ The term “quality of life” refers to a multifaceted term that includes various aspects related to overall wellbeing.^1^,^3^ Cardiovascular diseases are the major cause of morbidity and mortality in patients of T2DM and largest contributor to the direct and indirect costs of diabetes.^5^ According to previous literature almost 80% of End-Stage Renal Disease(ESRD) are also caused by T2DM.^7^ Despite advance treatment, complete cure of T2DM cannot be achieved so far, hence these patients remain anxious about their treatment and its cost.^3^

According WHO definition, “health is a state of complete physical, mental and social wellbeing, and not merely the absence of disease or infirmity”.^8^ High morbidity rate and fear of not getting cure of T2DM, compromise their physical, and social well-being that refers to poor QOL in these subjects.^3^,^6^ Now a days assessment of the QOL in patients with T2DM is gaining attention with recent guidelines from the American Diabetes Association that emphasize on the need of “patient-centered” approach of the management of T2DM patients in terms of QOL, prevention of diabetic complications, and achievement of glycemic targets.^9^ The SF-36 questionnaire is a globally used validated standardized tool for assessing general health of patients representing QOL.^10^ It emphasizes the patient’s perspective on health, which is crucial in chronic conditions like diabetes where self-management plays a significant role. Additionally, its comprehensive nature allows for the evaluation of various dimensions of physical and mental health, making it an ideal choice for understanding the impact of diabetes on patients’ overall well-being.^2^ This breadth allows for a holistic view of how diabetes affects patients’ lives. Moreover, it is sensitive to changes in health status, making it useful for tracking the progression of diabetes and the effectiveness of interventions over time. As a standardized tool, the SF-36 facilitates comparisons across different populations and studies, enhancing the generalizability of findings related to diabetes management and quality of life. SF-36.^2^

However, there is paucity of data for Pakistani population concerning QOL of T2DM patients. Hence the present study was aimed to address and analyze the impact of T2DM on QOL using Short Form-36(SF36) questionnaire and compare it with non-diabetic healthy subjects, so important measures could be taken to improve patients’ care and quality of life.

## METHODS

This case control study was the joint project of University Sains Malaysia (USM), Malaysia and Aziz Fatimah Medical and Dental College (AFMDC) Faisalabad, Pakistan.

### Ethical Approval:

It was approved by Research Review Board and Ethics Committee of USM, Malaysia (USM/JEPeM/21090630, dated April 5, 2023), and the Institutional Ethical Committee of AFMDC (Ref.no: IEC 123/21, dated May 24, 2021) which complies with Declaration of Helsinki.

### Inclusion & Exclusion Criteria:

This study was conducted at Aziz Fatimah Hospital (AFH) and AFMDC Faisalabad, Pakistan from May 2023 to January 2024. Patients of T2DM of both genders with age ranged 30 to 65 years, who fulfilled one of the following criteria of American Diabetic Association for diagnosis of T2DM were included in the study, criteria were as follow, FBS **≥ 126 mg/dl** on 2 more occasions, Glycated Hemoglobin (HbA1c) > 6.5% or Two hours post glucose load (75 gm) plasma glucose Plasma glucose ≥200 mg/dl. Age and sex matched healthy normal subjects were included as control group. Subjects with other endocrine diseases, cardiopulmonary diseases, Cancer patients, subjects diagnosed with mental disorders were excluded. Additionally, the subjects who did not filled the questionnaire completely were also excluded from the study. Sample size calculated was 172 participants (86 cases and 86 controls). Eighty-six diagnosed T2DM patients of both genders were enrolled from diabetic outpatient department of AFH using purposive sampling technique. Using same sampling technique, 86 Control subjects were enrolled from employees working at AFMDC and AFH. Two control participants have submitted incomplete proforma so we excluded them and finally 84 Control subjects were included. Hence, final data for 170 participants were analyzed. Informed consent was obtained from each participant, and confidentiality was assured. Fasting and random blood glucose was estimated by enzymatic colorimetric test (Glucose Liquicolor). HbA1c was determined by using Bioherms A1C EZ 2.0 Glyco-hemoglobin Test Kit.

QOL of patients with T2DM and Control subjects was assessed using validated and reliable short form series -36 (SF-36) version -1 questionnaire in English with Cronbach’s alpha 0.85. SF-36 scale comprised of thirty-six items, of which thirty-five items were for assessing the eight subscales of physical and mental domains of health (four subscales for each physical and mental domains), while one item was about change in health status in past one year. Physical and mental domains are summarized in two summary measures including physical component summary (PCS) mental component summary (MCS) respectively. The physical domains included physical parameters such as physical functioning (PF); role limitations due to physical health problems (RP); bodily pain (BP) and general health perceptions (GH). Mental domains included vitality(VT), social functioning(SF); role limitations due to emotional problems(RE) and mental health(MH).^10^ Submitted responses of SF36 questionnaire for all eight subscales were transformed to a linear 0-100 scale to calculate raw scores as per RAND soring instruction.^11^ Higher scores, approaching 100 representing better QOL.

All Scores of physical and mental subscales were standardized using R studio software to calculate the PCS and MCS scores respectively according to the SF-36 physical and mental health summary scales user’s manual.^12^ The PCS and MCS domains were developed to simplify analysis by consolidating subscale scores, thereby enhancing reliability, validity, and the ability to discriminate between physical and mental health outcomes. Cutoff points were 50 for each PCS and MCS to evaluate QOL. Subjects having MCS and PCS scores below 50 considered QOL below average or impaired QOL.^10^ This questionnaire was free to use and no license was required to use this proforma.^13^

### Statistical Analysis:

Data was analyzed using SPSS26. Continuous variables were expressed as mean ±SD. Categorical variables were presented as frequency and percentages. The mean scores of all subscales of SF36 and PCS and MCS were compared between T2DM and Control groups using the independent t-test implying central limit theorem as sample size was greater than 30, therefore parametric test was used. Mean of all scores were also compared by the status of gender and duration of T2DM. p ≤0.05 is considered statistically significant.

## RESULTS

The study was comprised of total 170 participants with mean ±SD of age 49.23 ± 12.59 years. Out of total population, 86(50.6%) were T2DM patients as cases and 84(49.4%) were normal healthy subjects as Control group. Regarding gender, 76(44.7%) were males and 94(55.3%) were females. Glycated hemoglobin (HbA1c), fasting and random blood sugar levels were significantly higher in T2DM patients as compared to control group (Table-I).

**Table-I:** Biochemical Profile of the Study Participants Comparison of Glucose Indices between Type-2 Diabetes Mellitus and Control Groups (n=170).

Glucose Indices	Control Group (n=84)	T2DM Group (N=86)	P value
	Mean ± SD	Mean ± SD	
Random blood Sugar (mg/dL)	108.58±51.01	175.74±187.50	0.000*
Fasting Blood Sugar (mg/dL)	86.34±17.90	180.56±62.55	0.0001*
Glycated hemoglobin (HbA1c))	5.28±50.53	8.14±1.82	0.000*

SD; standard deviation, mg/Dl; milligrams per deciliter, Independent t test was used to compare the mean,

* Statistically Significant value at P ≤ 0.05.

Current results showed significant lower scores of subscales of physical domain including physical functioning (p value= 0.003), limitation of activities due to physical health (p value =0.050) and bodily pain (p value =0.030) in T2DM patients in contrast to Control subjects (Table-II). Interestingly, T2DM group had significantly higher general health perception scores as compared to the Control group, indicating T2DM subjects have comparatively better general health perception than the Control group (p =0.000) (Table-II).

**Table-II:** Comparison of Mean Scores of SF-36 Domains between Type-2 Diabetes Mellitus and Control Groups (n= 170).

SF-36 Subscales and Summary Measures	Control Group n (%) 84(49.4)	T2DM n (%) 86 (50.6)	P value
	Mean ±SD	Mean ±SD	
Physical Functioning	62.38 ± 26.70	50.36±24.59	0.003*
limitation of activities due to physical health	45.23 ±38.899	34.01±36.36	0.050*
limitation of activities due to emotional problem	44.04 ± 39.18	26.68±30.21	0.001*
Social Functioning	63.21 ± 18.88	59.87± 18.47	0.245
Bodily Pain	57.90± 19.19	51.22±20.59	0.030*
General Health	36.19±11.55	53.54± 14.90	0.000*
Vitality	57.58±15.96	45.29±15.96	0.000*
Mental Health	64.33±18.85	56.79± 18.85	0.009*
Physical Component Summary (PCS)	38.47±6.73	37.75±7.55	0.511
Mental Component Summary (MCCS)	44.29±9.39	40.04±8.10	0.002*
Change in Health Status in One Year	48.80±20.86	45.63±28.23	0.406

SD; standard deviation, T2DM; Type-2 Diabetes Mellitus, Independent t-test was used to compare mean,

* Statistically Significant value at P ≤ 0.05.

Present results revealed similar pattern in subscales of mental domain indicating significantly lower score for vitality indicating a low level of energy and higher tendency to fatigue in the T2DM patients than Control subjects, (p= 0.000), (Table-II). Significantly higher score for mental health in the Control group stated that these subjects were more mentally stable and had less psychological distress in contrast to T2DM patients (p =0.009). However, no significant difference was found in term of score for social functioning assessing how much emotional problems interfered with the normal social activities with family, friends or neighbors etc. (p = 0.245), (Table-II).

Two summary measures, Physical component summary (PCS) and Mental component summary (MCS) scores reflecting overall quality of life were below 50 in both T2DM and Control groups indicating poor physical and mental health outcomes in both groups (Table 2.1). No significant difference was found in PCS scores among Control and T2DM groups (p=0.511), however MCS scores were significantly higher in Control group (p=0.002), (Table-II) change in health status in one year is an additional parameter other than eight subscales of physical and mental domains, reflecting how much change in overall health status they observed in last one year, was not significantly different among the T2DM and Control group (p=0.406), (Table-II).

Current results showed that T2DM > 5 years have comparatively higher scores than T2DM< 5 years with respect to physical functioning (PF), limitation due to physical health (RP), vitality (VT), mental health (MH) and social functioning (SF), however the difference was statistically significant only for VT subscale of mental domain indicating T2DM >5Year had more energy and resistance to fatigue as compared to shorter duration of diabetes (p=0.004). On contrary, shorter duration of T2DM have comparatively higher bodily pain (BP) scores indicating pain was more pronounced in patients with T2DM < 5yeas duration when compared with longer durationT2DM (Table-III).

**Table-III:** Comparison of Quality of Life by the Status of Duration of Type-2 Diabetes Mellitus(n=86).

SF-36 Subscales and Summary Measures	T2DM< 5 Year n (%) 40(23.5)	T2DM >5 Year n (%) 46 (27.1)	P value
	Mean ±SD	Mean ±SD	
Physical Functioning	48.60 ± 19.93	52.32 ± 29.19	0.482
limitation of activities due to physical health	33.15 ± 34.58	35.00 ± 38.72	0.816
limitation of activities due to emotional problem	31.82 ± 29.02	20.77 ± 30.83	0.091
Social Functioning	58.67 ± 16.94	61.25 ± 20.21	0.522
Bodily Pain	54.86 ± 18.02	47.02 ± 22.70	0.078
General Health	53.16 ± 14.38	54.00 ± 16.65	0.794
Vitality	43.95 ± 16.00	46.82 ± 15.99	0.004*
Mental Health	55.95 ± 18.67	57.75 ± 19.25	0.663
Physical Component Summary	37.53 ± 7.42	38.00 ± 7.78	0.773
Mental Component Summary	40.28 ± 7.65	39.77 ± 8.69	0.775
Change in Health Status in One Year	44.56 ± 28.33	46.87 ± 28.41	0.707

T2DM; Type-2 Diabetes Mellitus, SD; standard deviation, Independent t-test was used to compare mean,

* Statistically Significant value at P ≤ 0.05.

PCS and MCS scores were below 50 in both shorter and longer duration of T2DM patients reflecting poor quality of life in both groups. Mean scores were not significantly different for longer and shorter duration T2DM with p values = 0.773 and 0.775 respectively. (Table 4.2). Scores for change in health status in one year was lower in both shorter and longer duration of T2DM indicating patients in both groups felt that their health deteriorate for last one year(p=0.707).

Concerning gender differences, it was noticed that females have significantly lower scores in terms of vitality (VT), bodily pain (BP) and change in health status during one year as compared to males. Significant lower scores for VT in female indicated that females have low level of energy and ability to fatigue when compared to males (p =0.005). Significant lower scores for BP in females indicated that they significantly experienced more pain as compared to males (p = 0.031), (Table 2.3). Rest of the subscales and summary scores including PCS and MCS were not statistically significant as p value were > 0.05 (Table-IV).

**Table-IV:** Comparison of Quality of Life by the Status of Gender (n=170).

SF-36 Subscales and Summary Measures	Male n (%) 76(44.7)	Female n (%) 94 (55.3)	P value
	Mean ±SD	Mean ±SD	
Physical Functioning	55.00 ± 27.05	57.35 ± 25.73	0.564
limitation of activities due to physical health	45.72 ± 37.50	34.57 ± 37.76	0.057
limitation of activities due to emotional problem	37.65 ± 36.30	33.32 ± 35.65	0.436
Social Functioning	55.38 ±17.00	48.11 ± 16.01	0.005*
Bodily Pain	61.86 ± 20.03	59.42 ± 0.24	0.407
General Health	61.32 ± 19.72	61.68 ± 17.93	0.903
Vitality	58.23 ± 20.40	51.52 ± 19.50	0.031*
Mental Health	43.15 ± 16.80	46.43 ± 15.07	0.182
Physical Component Summary	38.53 ± 7.85	37.76 ± 6.53	0.488
Mental Component Summary	43.05 ± 10.07	41.41 ± 7.99	0.240
Change in Health Status in One Year	53.28 ± 24.94	42.28 ± 23.76	0.004*

SD; standard deviation, Independent t-test was used to compare mean,

* Statistically Significant value at P ≤ 0.05.

## DISCUSSION

In present study quality of life of T2DM patients was assessed and compared to Control group using SF36 questionnaire. Results of the current study showed that T2DM patients have lower mean scores than the Control group participants in-term of physical functioning, role limitations due to physical health, role limitations due to emotional problems, vitality reflecting energy/fatigue levels, mental health, bodily pain and general health subscales of physical and mental domains of health. Two summary measures PCS and MCS representing the summary scores of physical and mental domains of health indicated the status of quality of life (QOL) depending on cut of points of 50 for each PCS and MCS.

Although the mean PCS and MCS scores were below 50 in both Control and T2DM groups, but former showed worst results. These scores of < 50 showed poor QOL in both groups. Present study concerning QOL in Control healthy subjects supports the findings of the prior study conducted in Abbottabad , Khyber Pakhtunkhwa , Pakistan, that found overall, lower QOL scores in terms of physical ,psychological, social relationship and environmental health domains in Pakistani general population when compared to many other developing countries.^14^ The justification for poor QOL in Control group might be due to the declining economy, and skyrocketing inflation in Pakistan that has exacerbated the difficulties of meeting their daily expenses. This all along with the social pressure leading to anxiety and psychological problems causing devastating impact on QOL of Pakistanis across all income groups and everyday life for them has become a desperate struggle for survival.^14^,^15^

The current findings regarding the QOL of patients with T2DM are corroborated by a study conducted in Peshawar, Pakistan which reported a poor QOL among T2DM patients^16^ These findings are consistent with the recent study conducted by Esubalew et al in Ethiopia reported poor QOL of T2DM patients and suggested the presence of co-morbidities with T2DM further deteriorates QOL represented by physical and mental domain and summary scales including PCS and MCS scores.^17^ According to this aforementioned study most of the uncontrolled T2DM patients required insulin injections for glycemic control, fear of self-administration of Insulin injection is also one of the important contributing factors for impaired QOL in T2DM patients.^17^ Interesting findings have been documented by Verhulst et al study conducted in the Netherlands on the Dutch population, they reported significantly lower SF36 scores for each domain among the T2DM patients with impaired oral health than general healthy population from the Netherlands. However, T2DM patients without an impaired oral health, had comparable or even higher SF-36 scores in all domains except for physical functioning and general health perceptions than the healthy population.^18^

Poor QOL may be attributed to the participants’ insufficient self-discipline in terms of diet and physical activity and adoption of more sedentary life styles. Moreover, the lack of standard health care services and guidance for patients in underdeveloped areas may have resulted in the early development of complications leading to poor QOL.^17^ Recent study conducted in Peshawar has documented that malnutrition in diabetic patients is a contributor for early development of complications and prolong hospitalization leading to increase in overall cost of treatment. This could increase burden not only on the patients but also on their caretakers, impairing their QOL.^19^

Furthermore, previous studies also show that socio-demographic factors could also contribute to the observed differences in QOL among the patients of T2DM and Control group.^15^ Current results were in line with, Sendekie et al, who reported compromised QOL of majority of the T2DM patients. This aforementioned study has observed overall lower scores of QOL in T2DM patients as compared to general healthy population.^20^ Sendekie et al and colleagues inferred from their findings that compromised QOL in T2DM patients attributed to poor glycemic control, use of multiple medicines specifically insulin and the development of complications. Moreover, unaffordability to purchase expensive multiple medications is additional contributing factors to worsen their QOL.^20^

The current study results were also in line with Malaysian study documented lower scores of QOL in T2DM patients.^21^ Interesting finding have been reported by this aforementioned study, that T2DM patients with good glycemic control reported more negative impact on QOL as compared to poor glycemic control. A possible reason for this fact was that the patients conscious about their health always remain anxious about their diet and daily activities following hard lifestyles to achieve desired glycemic control. These patients were subjected to strict management and needed regular medical check-ups, and costly medicines, adding another burden to these individuals especially those who were not financially sound.^21^ Fear of disabilities such as, retinal detachment leading to blindness, and diabetic foot syndrome leading to amputation of lower limbs, is also the key factor associated with the negative impact of T2DM on QOL.^21^,^22^ Present results indicate that the subjects with T2DM experienced bodily pain more profoundly than normal healthy subjects. These results were supported by a previous study stating that pain followed by depression and anxiety, were the most common associated problems related to T2DM and it negatively affects patients’QOL.^23^ Present results were supported by a previous study conducted in Saudi Arabia that reported higher prevalence of chronic pain involving multiple sites in subjects with diabetes, the author of this aforementioned study had suggested that chronic pain is a comorbid ailment, that can be troublesome in T2DM patients due to symptomatic manifestations along with psychological and physical disability, which limits their routine self-care and affect the quality of life.^24^

Current results concerning impact of duration on QOL are not generally correspond with reporting from previous studies. In the previous studies long duration of diabetes was identified as a significant predictor of QOL predictor.^17^,^21^ Surprisingly we observed that patients having longer duration T2DM > 5 years have comparatively higher scores with respect to physical functioning (PF), limitation due to physical health (RP), vitality (VT), Mental health (MH) and social functioning (SF). Higher scores for VT in patient’s longer duration of T2DM showed that they were more energetic and resistance to fatigue as compared to shorter duration of T2DM. Similarly, Higher scores for SF and MH in patients with longer-duration diabetes indicates that they were more socially active with their families and relatives and mentally stable and had less psychological distress despite the long duration of diseases. Contradictorily, a study conducted in Pakistan found no significant association between QOL and the duration of diabetes mellitus, suggesting that this may be due to physical and mental adaptation to the chronic disease process.^16^

Numerous previous studies reported that longer duration of T2DM for more than 10 to 15 years, had significant negative impact on QOL.^17^,^21^ The possible logistic reason explained by the previous studies in above context may be that the glycemic control tends to be worse with a longer duration of diabetes due to a deterioration in beta cell function and resultant decrease in insulin secretion leading to vicious cycle causing more worsening of glycemic control and metabolic dysregulation.

However, it was inferred from present study results that lower scores for QOL in short duration of T2DM as compared to longer duration of T2DM may be due to the fact that the newly diagnosed patients often experience denial and exhibit negative attitude towards the aggressive treatment regimen and avoidance of medication may interfere with attaining desired glycemic control leading to worsening their condition and devastating impact on their QOL.

Results of present study revealed lower scores of most domains of SF-36 except for physical functioning in females as compared to male counterparts reflecting poor QOL in them. These results were consistent with Pakistani study, in which women reported poorer QOL than men in social relation and psychological health domains in Pakistani general population.^14^ Similarly poor QOL was reported for T2DM female patients by another Pakistani study conducted by Sarir N at Peshawar Pakistan. 16 Present study findings agreed with recent study conducted in Iraq reporting lower scores of all domains of SF-36 in female in comparison male participants.^2^ Similar to current results, worse QOL in females than males were reported by the study conducted in Spain.^25^ This Spanish study has suggested that women’s worse QOL was due to a higher prevalence of reported chronic conditions and disability in them as compared to men.^25^ This explanation further strengthens present study results as most of the participants of the current study had chronic condition “T2DM”. Poorer scores of QOL in females warranted physical, social and psychological support for females to improve their QOL.

Understanding the impact of T2DM on QOL is imperative for management of this chronic condition. Along with the clinical management, psychosocial support and self- care activities including lifestyle modifications such as strict dietary intervention combined with comprehensive exercise, weight reduction can help in achieving glycemic control and better health outcomes with improved QOL.^1^

Pakistan’s diverse ethnic groups face unique cultural, societal, and economic challenges that hinder self-care actions of diabetic patients negatively impacting their QOL. Psychosocial and cultural obstacles for self- care include negative thoughts about diabetes and its cure, family ignorance, negative peer pressure, financial constraints, feelings of being a burden on families, limited access to healthcare services, low literacy levels in rural area, and cultural attitudes toward diabetes further complicate management and leading to emotional problems in diabetic patients. 26 To overcome these challenges, psychosocial support programs and diabetes education are crucial, and there is an urgent need to incorporate psychosocial care as a routine part of diabetes management in Pakistan, where it is currently lacking. Positive impact of social capita on QOL have been reported by previous Pakistani study involving huge sample of Pakistani general population of Khyber Pakhtunkhwa, province, Pakistan.^14^ However, now there is a need to understand its importance for patients with diabetes mellitus in Pakistan. Recently, several organizations have emerged to raise awareness about diabetes, providing hope for better management of its psychosocial aspects, though more efforts are needed. This all will reduce the overall healthcare cost and improve disease outcomes and quality of life.

### Strength of the study:

This study uniquely examines the quality of life (QOL) of T2DM patients in Pakistan using the validated SF-36 questionnaire. The findings of this study add to the existing medical literature by providing a comprehensive understanding of the QOL of T2DM patients in Pakistan, highlighting the physical and emotional burdens associated with this chronic condition. Present study emphasizes a patient-centered management approach, highlighting the significance of lifestyle modifications such as dietary changes and exercise in achieving optimal glycemic control. These changes can reduce reliance on expensive medications like insulin and alleviate the anxiety associated with their use, ultimately improving patients’ QOL. By shedding light on the physical and emotional challenges faced by T2DM patients, this research underscores the importance of holistic care in clinical practice, urging healthcare providers to prioritize overall well-being alongside blood sugar management.

### Limitation and Recommendations:

This study was single centered approached, so results may not be generalized to whole population. The study’s case-control design limits causal inference. Longitudinal studies could better elucidate the trajectory of QOL changes over time. Furthermore, quality of life can also be affected by the presence of complications of diabetes, socioeconomic status, psychosocial support, glycemic control levels, comorbidities as well as use of insulin vs oral hypoglycemic agents. These factors are not taken into consideration in present study which should be considered in future studies to strengthen the present results and to understand their specific contributions to QOL.

## CONCLUSION

Type-2 diabetes mellitus significantly impairs quality of life, particularly for females and those with a disease duration of less than five years’, who experience a greater negative impact as indicated by lower SF36 Scores.
